# Primary malignant melanoma of the esophagus treated with subtotal esophagectomy: a case report

**DOI:** 10.1186/s12893-017-0326-7

**Published:** 2017-12-02

**Authors:** Shota Kuwabara, Yuma Ebihara, Yoshitsugu Nakanishi, Toshimichi Asano, Takehiro Noji, Yo Kurashima, Soichi Murakami, Toru Nakamura, Takahiro Tsuchikawa, Keisuke Okamura, Toshiaki Shichinohe, Satoshi Hirano

**Affiliations:** Department of Gastroenterological Surgery II, Hokkaido University Graduate School of Medicine, North 15 West 7, Kita-ku, Sapporo, Hokkaido 0608638 Japan

**Keywords:** Esophagus, Melanoma, Pathology, Treatment, Prognosis

## Abstract

**Background:**

Primary malignant melanoma of the esophagus (PMME) is a rare disease with a poor prognosis. There are few reports of early-stage cases in which tumor invasion reached the lamina propria or muscularis mucosae, as in the present case. A standard treatment for early-stage PMME has not yet been established. The present study aimed to summarize previous reports and to discuss the indications for surgical treatment of early-stage primary malignant melanoma of the esophagus.

**Case presentation:**

A 70-year-old woman with PMME was referred to our hospital. She underwent thoracoscopic and laparoscopic subtotal esophagectomy with lymphadenectomy. The resected specimen showed melanocytosis and junctional activity. Melanoma-specific antigens melan-A, S-100, and HMB45 were detected by immunohistochemical staining. The pathological diagnosis was pT1a-MM, pN0, pM0, and pStage IA. She remains alive without evidence of recurrence 39 months later.

**Conclusion:**

Subtotal esophagectomy with regional radical lymphadenectomy could be recommended to patients with early-stage primary malignant melanoma of the esophagus, and curative surgical resection could improve their prognosis.

## Background

Primary malignant melanoma of the esophagus (PMME) is a rare disease. The incidence of PMME in all esophageal malignancies is low at 0.1%–0.2% [1]. The prognosis of PMME is poor because of its highly malignant biological behavior and its tendency to frequently disseminate even at the time of diagnosis. The recently reported 5-year survival rate after surgical resection is 37.5% [2], which is lower than that of esophageal cancer. Given that PMME is a rare disease with a poor prognosis, an appropriate treatment of choice for PMME is still under investigation. Here, we present a case of early-stage PMME in which tumor invasion reached the muscularis mucosae that followed a favorable course after subtotal esophagectomy.

## Case presentation

A 70-year-old woman presented at her local hospital with a sticky sensation in her throat and a weight loss of 2 kg over 10 months.

Esophagogastroduodenoscopy (EGD) revealed an elevated lesion 35 cm from the incisors that was diagnosed as malignant melanoma by biopsy. She was referred to our institution for further examination and treatment. Her blood examination was normal, including tumor markers such as CEA and CA19–9, except for a slightly elevated HbA1c level.

**Fig. 1:**
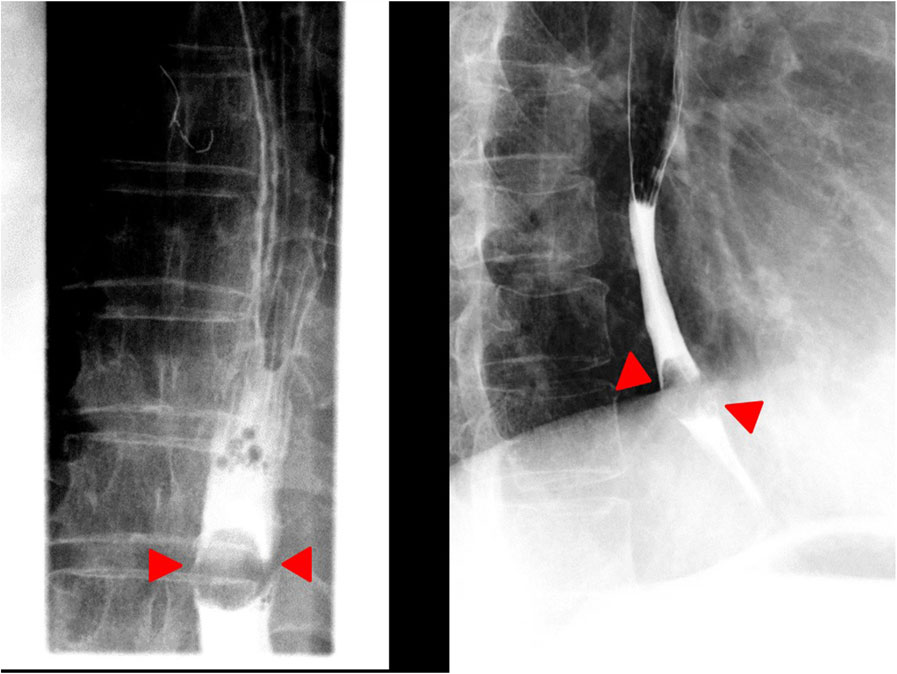
Findings of a barium swallow test. A barium swallow test shows a filling defect in the lower esophagus (arrows)

**Fig. 2:**
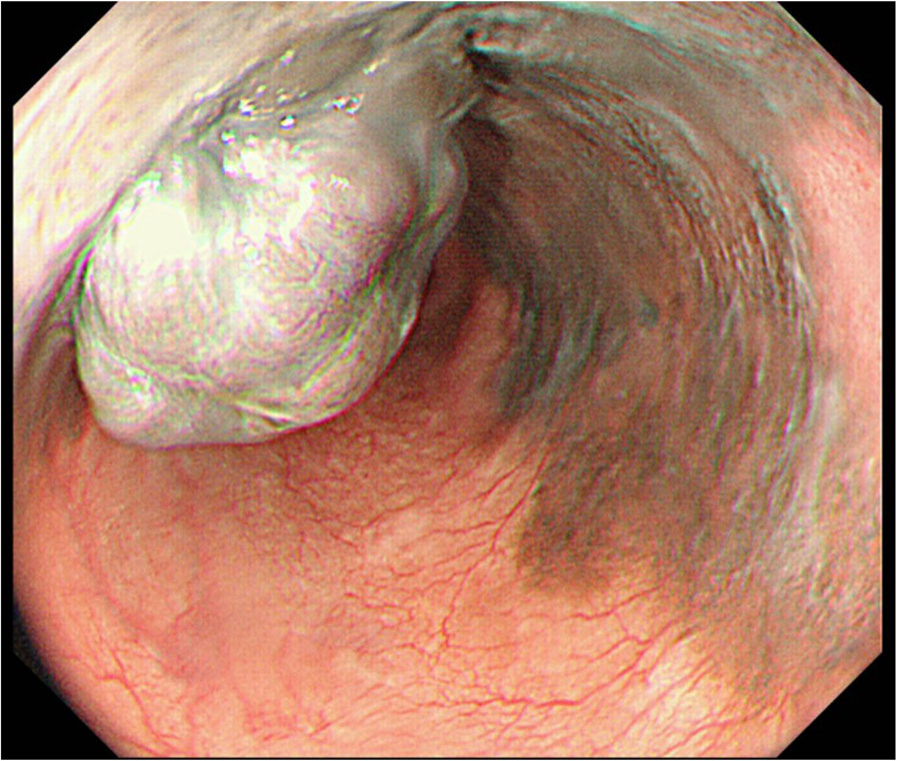
Findings of esophagogastroduodenoscopy (EGD). EGD shows a pigmented, elevated 7-mm lesion, indicating an almost circumferential, irregular-shaped pigmentation in the lower esophagus, ranging from dark brown to black. The black area contains a well-demarcated mucosal abnormality

A barium swallow test was arranged and showed a filling defect in the lower esophagus (Fig. 1). EGD showed a pigmented and elevated lesion 7 mm in diameter, associated with a hemi-circumferential, irregular-shaped, pigmented, and flat lesion in the lower esophagus. The flat lesion ranged from dark brown to black in color, and the black area contained a well-demarcated mucosal abnormality (Fig. 2).

**Fig. 3:**
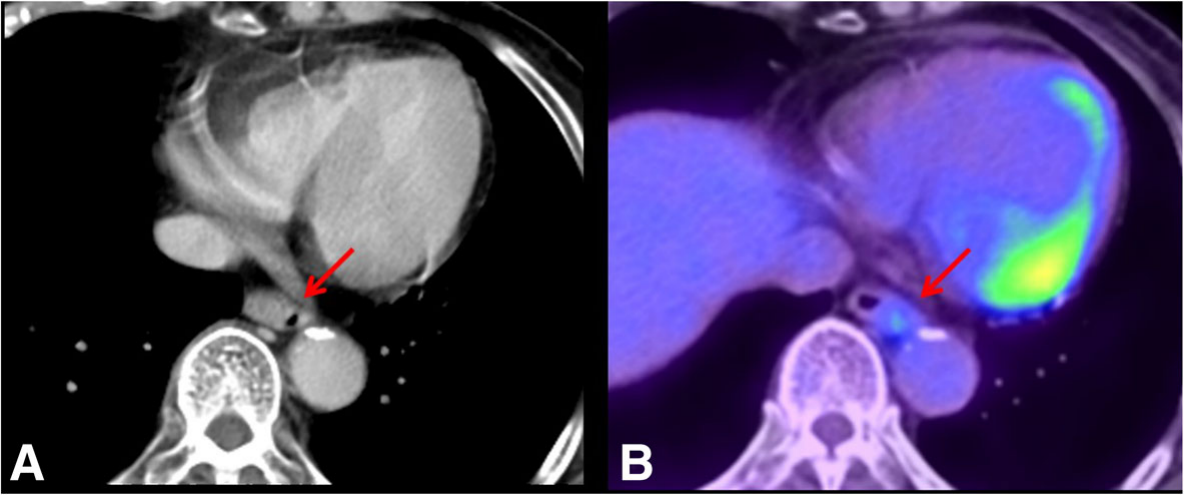
Findings of computed tomography (CT) and 18F–fluorodeoxyglucose (18F–FDG) positron-emission tomography/computed tomography (PET-CT). **a** A transverse plane of an enhanced CT scan shows an intraluminal mass in the lower esophagus, which is well-defined without infiltration of the surrounding tissue (arrows). **b** 18F–FDG PET-CT scan shows 18F–FDG uptake at the same lesion of the lower esophagus; the SUV max of the lesion is 3.1 (arrows)

A biopsy specimen showed malignant melanoma cells in the esophageal mucosa, which were strongly positive for melanoma-specific antigens S-100 and HMB45 by immunohistochemical staining. A computed tomography (CT) scan also showed an intraluminal mass in the lower esophagus, which was well-defined without infiltration into the surrounding tissues (Fig. 3a). There was no enlargement of mediastinal lymph nodes or any visible metastatic lesion. 18F–fluorodeoxyglucose (18F–FDG) positron-emission tomography (PET) combined with CT showed abnormal 18F–FDG uptake in the same part of the esophagus identified on EGD and barium swallow as the site of the lesion with a maximum standardized uptake value (SUV max) of 3.1 (Fig. 3b). Detailed clinical examination of the eyes, oral cavity, nose, and skin did not indicate any malignant melanoma lesions.

Based on these findings, the preoperative diagnosis of the lesion was PMME without metastasis (cT2N0M0, cStage II) according to the UICC TNM classification of esophageal cancer [3]. Then, thoracoscopic and laparoscopic subtotal thoracic esophagectomy with lymphadenectomy of the neck, mediastinum, and abdomen was performed. The cervical esophagus and the elevated gastric tube were anastomosed via the posterior mediastinal approach. The operation time was 536 min, and blood loss was 95 mL.

**Fig. 4:**
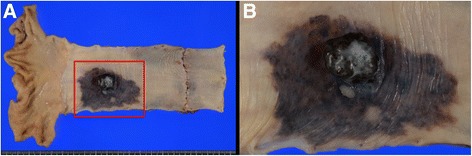
Macroscopic findings. **a** The resected specimen showed an elevated, black-pigmented polyp-like lesion (15 mm × 13 mm × 9 mm) on a flat, black-pigmented area (57 mm × 38 mm) in the lower esophagus. **b** A magnified image of the lesion (the part surrounded by a red square in Fig. 4a). There is a faintly marked depression partly tinged with white on the surface of the polyp-like lesion

**Fig. 5:**
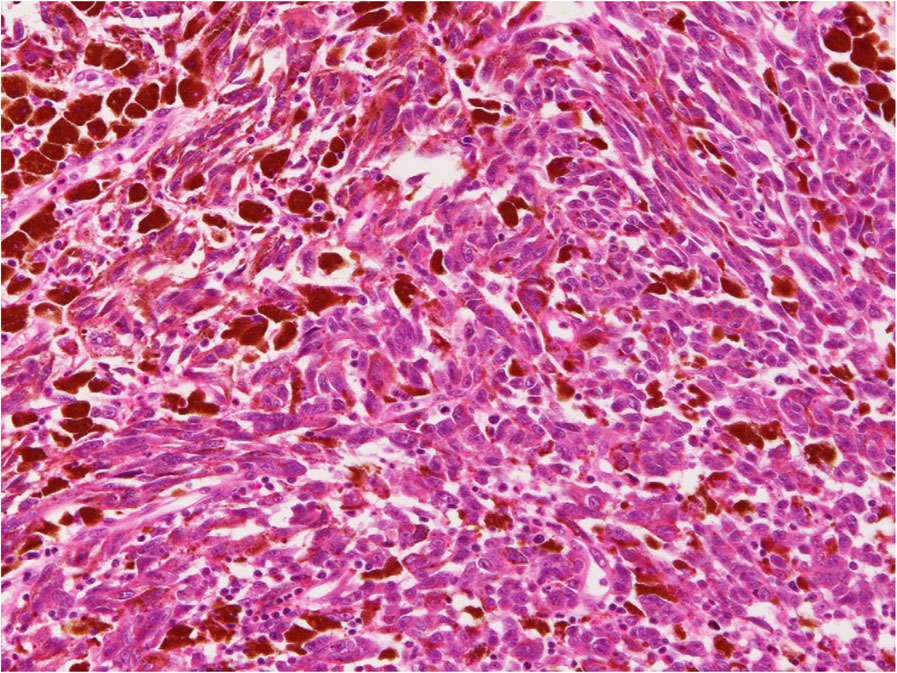
Histopathological findings (hematoxylin and eosin (HE) staining). Most of the polyp-like lesion is composed of solid growth, pseudocircular, and spindle-shaped atypical cells containing melanin pigmentation and irregularly demarcated nucleoli. Many melanophages are present in the intervening interstitial stroma (HE ×200)

**Fig. 6:**
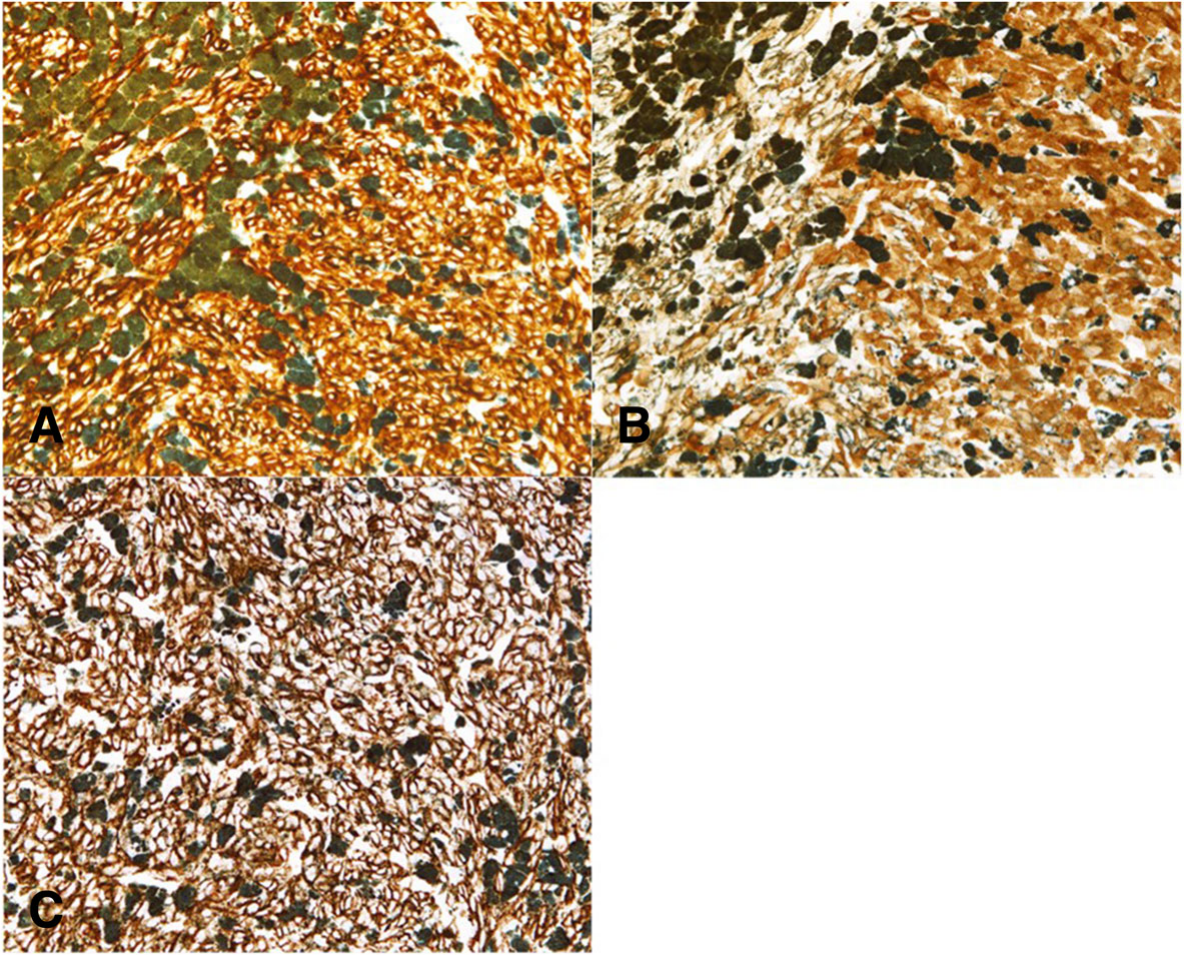
Histopathological findings (immunohistochemical staining). Melanoma-specific antigens melan-A, S-100, and HMB45 are shown by immunohistochemical staining. **a** Melan-A × 200, **b** S-100 × 200, **c** HMB45 × 200. The tumor cells are presented as brown pigment, and melanin is presented as green pigment

**Fig. 7:**
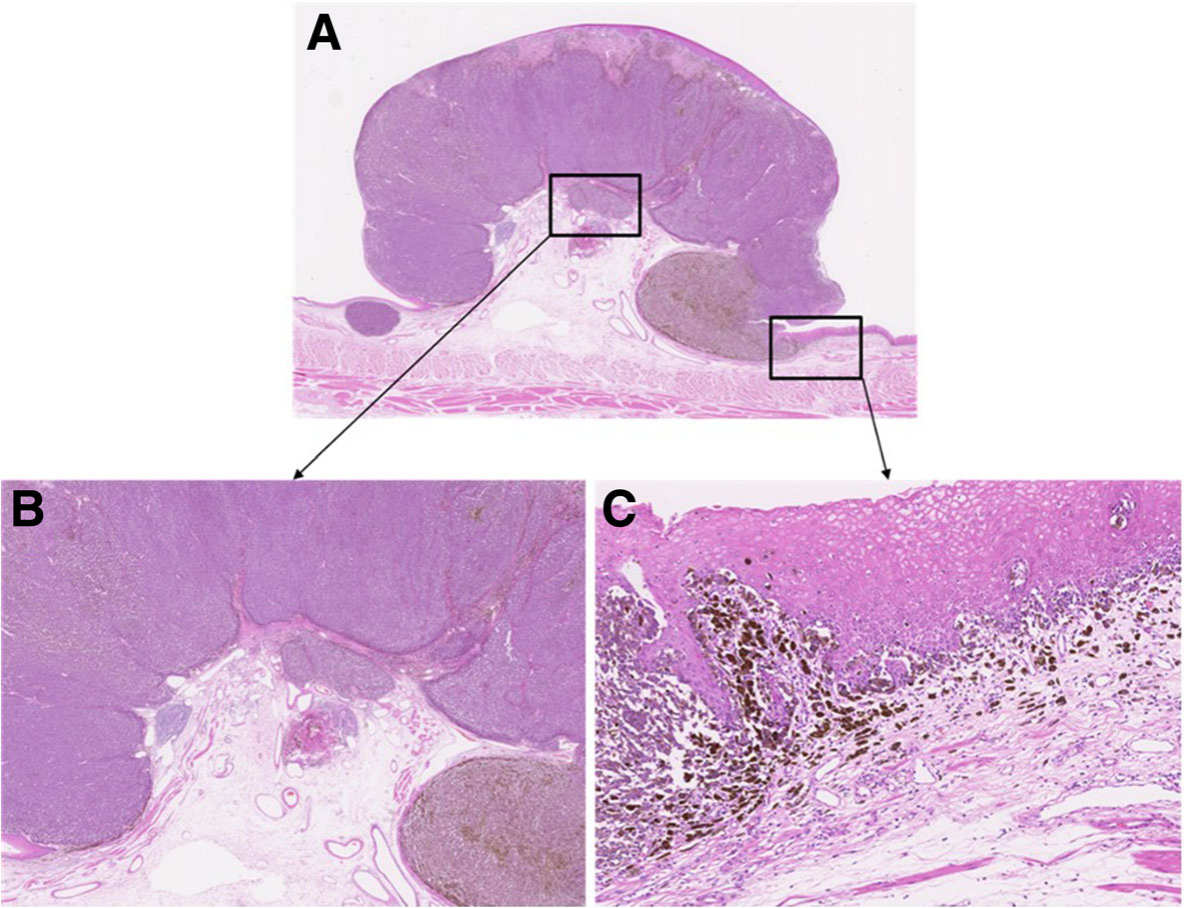
Histopathological characteristics of the PMME (hematoxylin and eosin (HE) staining). **a** The PMME shows a polyp-like intraluminal mass (HE ×10). **b** The tumor cell invasion directly involves the muscularis mucosae (HE ×25). **c** The tumor cells are spread horizontally in the basal layer of the esophageal epithelium, which is considered junctional activity (HE ×100)

The resected specimen showed an elevated, black, and pigmented polyp-like lesion (15 mm × 13 mm × 9 mm) in a flat and pigmented area (57 mm × 38 mm) (Fig. 4a). A faintly marked depression that was partly tinged with white on the surface of the polyp-like lesion was found (Fig. 4b). Microscopic examination showed that most of the polyp-like lesion was composed of solid growth, with pseudocircular and spindle-shaped atypical cells containing melanin pigmentation and irregularly demarcated nucleoli. Many melanophages were present in the intervening interstitial stroma (Fig. 5). The lesion was immunohistochemically stained strongly for melanoma-specific antigens melan-A, S-100, and HMB45 (Fig. 6a–c). Around the polyp-like lesion, the same characteristic cells were spread laterally in the epithelium. Melanophages were also present in the lamina propria beneath the polyp-like lesion. The tumor cells were thought to invade the muscularis mucosae directly and then spread horizontally in the basal layer of the esophageal epithelium, which is called “junctional activity” [4] (Fig. 7a–c). The proximal and distal margins were considered safe. No lymph node metastases were detected. Pathologically, a diagnosis of pT1a-MM, pN0, pM0, pStage IA [3] was rendered.

Her postoperative course was uneventful and favorable. Adjuvant chemotherapy was not administered, and she has survived 39 months so far without any evidence of recurrence. She has been followed up once a half year and underwent blood tests and contrast-enhanced CT to search for metastasis or recurrence.

## Discussion and conclusions

PMME is a rare disease with an extremely low incidence, comprising 0.1%–0.2% of all esophageal malignant tumors [1]. Over 70% of patients with PMME visit the hospital with chief complaints of dysphagia and epigastralgia [5]. Because the tumor is softer than other esophageal carcinomas, and wall extensibility of the esophagus is maintained, the onset of symptoms is slow despite the size of the tumor. Therefore, more than 90% of the tumors are found to be larger than 2 cm at the initial diagnosis [5], and the detection of PMME at an early stage is rare.

Generally, PMME tends to originate in the lower to middle esophagus with endoscopic findings of a well-circumscribed, elevated, and pigmented tumor that is partially covered by normal mucosa and rarely accompanied by ulcers. A black tone is well known as a characteristic of PMME, but various colors such as purple and brown are often present in 10%–25% of PMMEs depending on the melanin quantity [1, 6]. The diagnosis of PMME should be suspected when a black or dark brown mass is observed [7]; however, it is important to be aware of amelanotic melanoma without white melanin pigmentation. Therefore, careful assessment is necessary for an accurate diagnosis [6] by endoscopy.

Esophageal melanocytosis is characterized by the presence of an increased number of pigment-laden melanocytes in the basal layer of the esophageal squamous epithelium, and the transfer of melanin granules to the epithelium around the melanocytes [8]. It has been described as a premalignant lesion of PMME; therefore, differentiation from melanoma in situ is important [9]. A biopsy can be conducted on patients for definitive diagnosis, but its accuracy is only approximately 80% [5]. Moreover, 20%–50% of patients are misdiagnosed with a poorly differentiated carcinoma [5], especially in cases of amelanotic melanoma. Immunohistochemical investigations are supportive for definitive diagnosis [10].

Diagnostic criteria are defined by Allen and Spitz [4] as follows: (1) a typical histological pattern of melanoma, with melanin granules inside the tumor cells, and an (2) origin in an area of junctional activity in the squamous epithelium. Junctional activity is defined as some nests of melanocytes with varying degrees of atypia found at the mucosal-submucosal junction adjacent to the tumor mass [4]. In other words, the tumor cells are spread horizontally in the basal layer of the esophageal epithelium. These findings and the presence of in situ melanoma without previous history of cutaneous melanoma lead to the absolute diagnosis of PMME [10]. In the present case, melanocytosis and junctional activity were surrounding the main tumor, and positive results of melan-A, S-100, and HMB45 were revealed by immunohistochemical staining, which led to a definitive diagnosis of PMME.

The prognosis of PMME seems to be improving because of the advances in endoscopic technology. In 1989, Sabanathan [5] reported that 5-year survival rate of PMME after surgery was 4.2%, whereas Volpin et al. reported it was up to 37% in 2002 [11]. The increasing number of cases with early detection is one of the contributing factors of improving the prognosis [12]. However, the overall 5-year survival rate of advanced squamous esophageal carcinoma was reported to be 40%–50% [13], which is higher than PMME; therefore, the biological behavior of PMME appears to be aggressive. Invasion deeper than T2 (hazard ratio: 2.288, *p* = 0.0327, 95% CI: 1.071–4.878) [14] and lymph node metastasis (hazard ratio: 15.05, *p* = 0.013, 95% CI: 1.757–128.795) [15] have been reported as predictive factors for worse survival. On the contrary, Takahashi et al. [16] reviewed 33 patients with invasion depth of T1b, pointed out the poor prognosis of T1b patients because of a high recurrence rate (20 of 33 patients), and reported that the 5-year survival rate was only 29.4%.

Detection of the lesion in early stages seems to be relatively rare because of the characteristic delay in symptoms’ appearance. We identified 10 previous T1a cases [12, 15, 17–22] finally diagnosed by pathological tests, and their tumor location, size, depth of tumor, treatment, and outcomes were well-described. These cases were recorded from 1985 to 2015 and were found through literature search using the PubMed online database with “malignant melanoma” and “esophagus” as keywords (Table 1). In addition, no patients diagnosed in the early stage were reported in 1985–2000.

**Table 1:** Case reports of early primary malignant melanoma of the esophagus stage T1a (1985–2015)

Author	Year	Age	Sex	Location	Size (cm)	Depth^*a^	Treatment	Course^*b^
Kido [16]	2000	60	M	Lt	4.0 × 2.0	T1a	CR	33 months
Mikami [17]	2001	41	F	Mt	0.8 × 0.6	T1a	CR	31 months
Kimura [18]	2005	73	M	Lt	1.8 × 1.3	T1a-LPM	EMR	15 months
Suzuki [19]	2008	62	M	Mt	7.0 × 4.5	T1a-EP	CR	33 months
Suzuki [19]	2008	67	M	Lt	5.5 × 5.5	T1a-LPM	CR	53 months
Miyatani [11]	2009	64	F	Lt	0.5	T1a-LPM	EMR	20 months
Minami [20]	2010	72	M	Lt	unknown	T1a-EP	CR	25 months
Wang [14]	2013	62	M	Mt	7.0 × 4.5	T1a	CR	93.7 months
Yamamoto [21]	2015	75	M	Lt	1.5 × 1.0	T1a-MM	CR	83 months
Our case	2015	78	F	Lt	5.7 × 3.8	T1a-MM	CR	39 months

*^a^: According to the Japanese Classification of Esophageal Cancer, 11th Edition. Japan Esophageal Society Esophagus (2017). *^b^: All reported cases are still alive after the treatment, and none have had any symptoms of relapse or distant metastasis

*Mt* middle of the esophagus, *Lt* lower esophagus, *EMR* endoscopic mucosal resection, *CR* curative resection (subtotal esophagectomy and radical lymphadenectomy of the neck, mediastinum, and abdomen)

Among the 10 cases, subtotal esophagectomy with lymphadenectomy of the neck, mediastinal, and abdomen was performed in eight patients, and endoscopic mucosal resection (EMR) was performed in two patients because of the small tumor size. None of the cases received adjuvant therapy or had any signs of metastasis at diagnosis. Median disease-free survival time was 33 months and ranged from 15 to 94.7 months.

Treatment of PMME should be individualized for each patient. The choice should be based on tumor size and location, presence or absence of metastases, age, and comorbidities of the patients [23]. Kimura et al. [19] reported the first case of PMME treated by EMR and discussed the indications for EMR of superficial-type PMME. Miyatani et al. [12] reported that when the lesion was very small and a biopsy could not be obtained, EMR could be performed to obtain a definite diagnosis and to treat the patient. However, there have been few reports of PMME treated by EMR, and the indications for performing EMR should be evaluated cautiously after a detailed examination because of the risk of lymph node metastasis. Diagnosing PMME as T1a accurately is very difficult; therefore, we recommend subtotal esophagectomy with radical lymphadenectomy of the neck, mediastinum, and abdomen for treatment of PMME [D2]. Conversely, for patients with PMME at T1a, curative surgical resection could improve their prognosis. Although there is probably no absolute indication of adjuvant therapy for T1a and negative lymphoid metastatic cases because of low risk of metastasis and recurrence, as with esophageal cancer, careful follow-up such as blood tests including tumor marker and image inspection using contrast-enhanced CT would be necessary. On the contrary, systemic chemotherapy based on cutaneous malignant melanoma should be considered for deeper than T1b and positive lymphoid metastatic cases, but its efficacy in increasing overall survival of patients with PMME is still unknown. Meanwhile, neoadjuvant therapy for PMME has not been reported. Therefore, if the lesion was evaluated to be resectable at the time of diagnosis, curative surgical resection with radical lymphadenectomy of the neck, mediastinum, and abdomen [D2] could be performed.

We presented a rare case of early-stage PMME. A standard treatment for early-stage PMME has not yet been established, but subtotal esophagectomy with regional radical lymphadenectomy could be recommended for patients with PMME at T1a, and curative surgical resection could improve their prognosis. Further accumulation of cases is necessary to evaluate the relationship between treatment strategy and long-term prognosis.

## Abbreviations

CA19–9: Carbohydrate antigen 19–9
CEA: Carcinoembryonic antigen
EGD: Esophagogastroduodenoscopy
EMR: Endoscopic mucosal resection
PMME: Primary malignant melanoma of the esophagus
